# Longitudinal telomere length and body composition in healthy term-born infants during the first two years of life

**DOI:** 10.1371/journal.pone.0246400

**Published:** 2021-02-02

**Authors:** Kirsten S. de Fluiter, Veryan Codd, Matthew Denniff, Gerthe F. Kerkhof, Inge A. L. P. van Beijsterveldt, Laura M. Breij, Nilesh J. Samani, Marieke Abrahamse-Berkeveld, Anita C. S. Hokken-Koelega

**Affiliations:** 1 Department of Pediatrics, Subdivision of Endocrinology, Erasmus University Medical Center – Sophia Children’s Hospital, Rotterdam, The Netherlands; 2 Department of Cardiovascular Sciences, University of Leicester, Leicester, United Kingdom; 3 NIHR Leicester Cardiovascular Biomedical Research Unit, Glenfield Hospital, Leicester, United Kingdom; 4 Danone Nutricia Research, Utrecht, The Netherlands; 5 Dutch Growth Research Foundation, Rotterdam, The Netherlands; University of Newcastle, UNITED KINGDOM

## Abstract

**Objective:**

Leukocyte telomere length (LTL) is one of the markers of biological aging as shortening occurs over time. Shorter LTL has been associated with adiposity and a higher risk of cardiovascular diseases. The objective was to assess LTL and LTL shortening during the first 2 years of life in healthy, term-born infants and to associate LTL shortening with potential stressors and body composition.

**Study design:**

In 145 healthy, term-born infants (85 boys), we measured LTL in blood, expressed as telomere to single-gene copy ratio (T/S ratio), at 3 months and 2 years by quantitative PCR technique. Fat mass (FM) was assessed longitudinally by PEAPOD, DXA, and abdominal FM by ultrasound.

**Results:**

LTL decreased by 8.5% from 3 months to 2 years (T/S ratio 4.10 vs 3.75, p<0.001). LTL shortening from 3 months to 2 years associated with FM%(R = 0.254), FM index(R = 0.243) and visceral FM(R = 0.287) at 2 years. LTL shortening tended to associate with gain in FM% from 3 to 6 months (R = 0.155, p = 0.11), in the critical window for adiposity programming. There was a trend to a shorter LTL in boys at 2 years(p = 0.056). LTL shortening from 3 months to 2 years was not different between sexes.

**Conclusion:**

We present longitudinal LTL values and show that LTL shortens considerably (8.5%) during the first 2 years of life. LTL shortening during first 2 years of life was associated with FM%, FMI and visceral FM at age 2 years, suggesting that adverse adiposity programming in early life could contribute to more LTL shortening.

## Introduction

Telomeres are noncoding repetitive DNA sequences at the end of chromosomes, protecting genomic DNA in maintaining stability [1]. Due to the inability of DNA polymerase to fully replicate the ends of chromosomes, telomeres shorten with each cell division, thus with increasing age. When telomeres are reduced to a critical length, cells enter a state of arrest (cell senescence) [2]. Telomere length can thus be used as a proxy of biological aging and mortality [3], although it is not the only biomarker of aging.

The shortening of LTL can be accelerated by multiple factors, such as inflammation, (oxidative) stress, obesity, toxins and radiation [4]. Shorter telomeres are associated with an increased risk for cardiovascular diseases (CVD), but it is uncertain if telomere length can be seen as prognostic marker for CVD [3].

A rapid rise in weight during early life has also been associated with an increased risk for adiposity and CVD in adulthood [5–9]. We have shown that a rapid rise in FM% SDS during the first 6 months of life, the critical window for adiposity programming, results in higher FM% trajectories during infancy [10]. No associations were found between body size at birth and LTL in adulthood [11], but it is yet unknown whether telomere length and its changes over time are associated with longitudinally measured body composition during infancy and the gain in FM% during the critical window for adiposity programming.

Until now, one other study has investigated leukocyte telomere length (LTL) longitudinally in healthy, term-born infants during the first two years of life [12], which is an important period for infant development [13]. This study, however, did not investigate longitudinal LTL in association with body composition. Some studies in infants and children measured TL in cord blood directly after birth [14–16] or in childhood [17]. Obtaining longitudinal values for LTL in healthy, term-born infants in early life in association with longitudinal body composition measures is important for clinical and research use. Various conditions and syndromes are linked to altered telomere length and adverse body composition, for example in infants born prematurely [18], small-for-gestational age [15] and infants with various syndromes [19].

The primary objective of this study was to investigate longitudinal telomere length from age 3 months to 2 years. Our secondary objective was to investigate associations of telomere length with potential influencing factors like gestational age, birth size and parity and with longitudinal body composition and abdominal fat mass during the first 2 years of life. We hypothesized that infants with more fat mass and particularly more visceral fat mass have more shortening in telomere length during the first 2 years of life.

## Materials and methods

### Study settings and subjects

The study population consisted of healthy, term-born infants, participating in the Sophia Pluto Study, a birth cohort study in Rotterdam area (The Netherlands). Between January 2013 and October 2019, infants were recruited from obstetric departments of regional hospitals and primary health care centers and detailed data on body composition and growth during early life were obtained. The Sophia Pluto Study obtained approval by the Medical Ethics Committee of Erasmus Medical Center and parental written informed consent was obtained for every participant.

All participants fulfilled the following inclusion criteria: term born (≥ 37 weeks of gestation), age < 28 days, uncomplicated neonatal period without signs of severe asphyxia (defined as an Apgar score < 3 after 5 minutes) and no sepsis or long-term complication of respiratory ventilation. Infants were excluded if they had known congenital or postnatal diseases, confirmed intrauterine infection, maternal use of corticosteroids during pregnancy or a significant maternal medical condition that could interfere with the study results.

### Data collection and measurements

Outpatient clinic visits were scheduled at age 1, 3, 6, 9, 12, 18 and 24 months (Table 1). Data on pregnancy and birth outcomes were obtained and measurements were performed by trained staff. If an infant was ill at time of a scheduled study visit, parents were instructed to contact the study team in order to reschedule the appointment.

**Table 1:** Clinical characteristics of boys and girls.

Age		1 month	3 months	6 months	9 months	12 months	18 months	24 months
**N [Male]**		145 [85]	145 [85]	145 [85]	145 [85]	145 [85]	145 [85]	145 [85]
**Weight (kg)**	M	4.39 [0.55]	6.31 [0.68]	8.12 [0.90]	9.44 [1.03]	10.34 [1.19]	11.78 [1.43]	13.18 [1.71]
	F	3.92 [0.62]	5.62 [0.75]	7.39 [0.83]	8.62 [0.96]	9.48 [1.00]	10.91 [1.15]	12.31 [1.26]
**Length (cm)**	M	55.0 [1.99]	62.2 [1.91]	68.9 [2.18]	73.7 [2.54]	77.3 [2.84]	84.0 [3.09]	89.9 [3.47]
	F	53.3 [2.51]	59.9 [2.47]	66.6 [2.33]	70.9 [2.53]	74.9 [2.68]	81.6 [2.58]	87.9 [3.08]
**FM (%)**	M	15.9 [4.31]	22.8 [4.75]	24.4 [4.95]	22.1 [5.55]	20.8 [4.81]	18.8 [4.57]	17.8 [4.10]
	F	16.0 [5.08]	23.1 [5.42]	24.6 [5.53]	24.9 [4.80]	21.8 [5.03]	19.0 [4.36]	18.6 [4.22]
**Abdominal subcutaneous FM (cm)**	M	NA	0.41 [0.11]	0.43 [0.11]	0.39 [0.10]	0.35 [0.11]	0.34 [0.11]	0.35 [0.11]
	F	NA	0.39 [0.12]	0.41 [0.11]	0.38 [0.10]	0.34 [0.10]	0.32 [0.11]	0.34 [0.10]
**Visceral FM (cm)**	M	NA	2.42 [0.62]	2.28 [0.64]	2.42 [0.60]	2.43 [0.68]	2.31 [0.66]	2.11 [0.56]
	F	NA	2.22 [0.58]	2.24 [0.56]	2.52 [0.62]	2.49 [0.59]	2.27 [0.63]	2.25 [0.54]

Data expressed as pooled means [pooled standard deviation of the mean] for boys (M) and girls (F). Abbreviations: N; number, FM; fat mass.

### Anthropometrics

Weight was measured with an electronic infant scale to the nearest 5 grams (SECA 717, Hamburg, Germany). Length was measured twice by two-person technique with an infantometer to the nearest 0.1 cm (SECA 416) and head circumference was measured twice as the widest frontal-occipital circumference with a measuring tape to the nearest 0.1 cm (SECA 201). Weight-for-length, weight-for-age and height-for-age SDS were calculated by Growth Analyser (https://growthanalyser.org/; Talma, 2010).

### Body composition measurements

Until age 6 months, body composition was assessed by air-displacement plethysmography (ADP by PEA POD, COSMED, Italy) as described in detail elsewhere [20]. According to standard protocol, the PEA POD was calibrated daily [21].

From 6 months onwards, a Dual Energy X-ray Absorptiometry (DXA) scan was performed at every visit with the same device (DXA, Lunar Prodigy, GE Healthcare, UK) and software (enCORE software version 14.1). At the transition point of 6 months, median FM% was 24.1 by ADP and 25.0 by DXA (n = 278), with a median difference of 0.9% between both measurements. Bland-Altman analysis showed no proportional bias (p = 0.321) [22].

Fat mass index (FMI) was determined by dividing fat mass (kg) by height squared (m^2^) and FFMI by dividing fat-free mass (kg) by height squared (m^2^).

### Ultrasound measurement of abdominal fat mass

Subcutaneous and visceral fat thickness were measured by ultrasound at every visit starting from 3 months of age and described in detail elsewhere [20, 23]. Unsuccessful ultrasound measurements of visceral fat mass, without visualization of the lumbar vertebra, were excluded from analyses.

### Infant feeding

Infant feeding was classified as exclusive breastfeeding (BF) if an infant received breastfeeding for at least 3 months or no exclusive breastfeeding if they received formula feeding or mixed feeding before age 3 months. Information on the timing of solid food introduction was obtained from questionnaires.

### Telomere length assessment

Genomic DNA was isolated from peripheral leukocytes using standard procedures and the same methods were used for all samples. All LTL measurements were made in the same laboratory at the University of Leicester. Mean LTL was measured by the quantitative PCR-based technique [2, 24]. Telomere sequence copy number (T) was compared with a single copy gene number in the genome 36B4 (S) and telomere length expressed as a T/S ratio. All T and S values were calculated relative to a calibrator DNA (genomic DNA from the K562 cell line) that was included on every plate. This allowed correction for inter-run variation. To further minimize any technical variation in the LTL measurements for the 3 month and 2 year samples, both samples for each individual were run within the same assay plate. All samples were checked for concordance between duplicate values for T and S as quality control. Samples showing a difference of greater than 0.2 cycles in the take-off value or amplifying outside of the linear range of the assay were excluded and re-run alongside the second time point for that individual. Reproducibility of the assay was tested by re-running samples on separate days. The mean inter-run CV for the T/S ratio was 2.85%. T/S ratio in our cohort at 3 months and 2 years were compared with T/S ratio in our PROGRAM study at 21 years. Subjects of our PROGRAM study met the same inclusion criteria of the healthy, term-born infants of present study and samples were analyzed in the same laboratory using the same technique [18].

### Statistical analysis

Clinical characteristics were expressed as median and interquartile range [IQR], and pooled means (pooled SD) in Table 1. For this study, we included 145 infants with blood collection at age 3 months and/or 2 years with ≥ 4 body composition measurements during the first 2 years of life and without past or present serious illnesses. In total, 112 infants had blood collection at both time points in which the shortening in LTL from 3 months to 2 years was determined. Differences in clinical characteristics were assessed by independent student’s t-test or Mann-Whitney U-test for non-parametric parameters. Correlations were determined by Spearman’s correlation coefficient.

Missing data on body composition, mainly because of infants showing resistance at measurements, were imputed using a multiple imputation approach in SPSS to generate 20 imputed datasets. Although small differences were observed between analyses with imputed missing data and complete cases only, the main interpretation of the results and conclusions of the results were similar.

SPSS statistical package version 25 (SPSS Inc. Chicago, Illinois) was used. P-values < 0.05 were considered statistically significant.

## Results

Clinical characteristics of the subjects are presented in Table 1. Of the total group, 58.6% was male and 41.4% female.

Median [IQR] birthweight was 3.38 [3.11–3.90] kg at 39.9 [39.0–40.9] weeks in boys and 3.19 [2.78–3.50] kg at 39.9 [38.8–40.5] weeks in girls.

### Telomere length during the first 2 years of life

**Fig 1:**
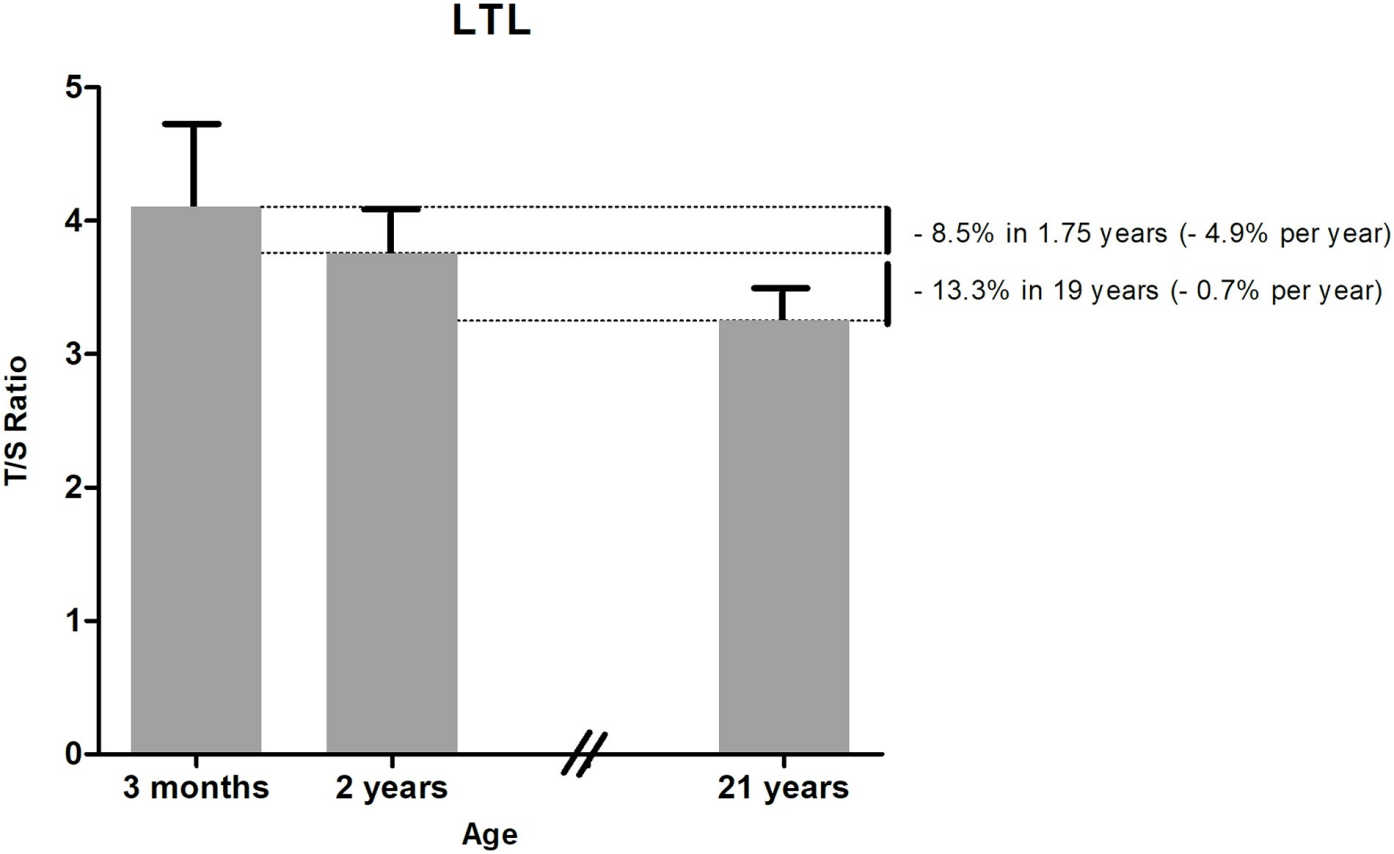
Longitudinal LTL development over time based on outcome of two separate birth cohort studies. Data are expressed as median LTL at 3 months and 2 years (current study) and mean LTL at 21 years (from our previous PROGRAM study [18]), both with upper interquartile range.

Median (IQR) LTL decreased from 3 months to 2 years (T/S ratio 4.10 (3.78–4.72) vs 3.75 (3.51–4.09), p<0.001) (Table 2), which is a decrease of 8.5% from 3 months to 2 years (4.9% per year) (Fig 1). LTL at 3 months was associated with LTL at 2 years (R = 0.641, p<0.001).

**Table 2:** LTL (T/S ratio) at age 3 months and 2 years for the total group, boys and girls.

	LTL 3 months	LTL 2 years	P-value
**Total group**	4.10 [3.78–4.72]	3.75 [3.51–4.09]	**<0.001**
**Boys**	4.05 [3.67–4.84]	3.65 [3.34–4.05]	**<0.001**
**Girls**	4.20 [3.86–4.67]	3.86 [3.53–4.29]	**<0.001**
**P-value**	p = 0.48	p = 0.056	

Data expressed as median [IQR]. Abbreviations: LTL; leukocyte telomere length, T/S ratio; Telomere to single-gene copy ratio.

### Telomere length and body composition during the first 2 years of life

The shortening in LTL from 3 months to 2 years associated with fat mass percentage (FM%, R = 0.193, p = 0.048), FM index (FMI, R = 0.243, p = 0.016) and visceral FM (R = 0.234, p = 0.022) at age 2 years, but not with abdominal subcutaneous FM. There was no association between LTL at age 3 months and 2 years and FM%, FMI, abdominal subcutaneous and visceral FM at the same ages.

As we previously found in the same study group that the gain in FM% from 3 to 6 months was associated with a higher FM% at 2 years, we investigated if the shortening in LTL from 3 months to 2 years was associated with the gain in FM% from 3 to 6 months. The shortening in LTL from 3 months to 2 years tended to associate with the gain in FM% from 3 to 6 months (R = 0.155, p = 0.11).

LTL at age 3 months, 2 years and the shortening in LTL from 3 months to 2 years did not associate with the change in FM% and visceral FM from 3 months to 2 years.

The shortening in LTL from 3 months to 2 years associated with fat-free mass index (FFMI) at 3 months (R = 0.223, p = 0.019), but not at age 2 years and not with FFM, and there was no association between LTL at age 3 months and 2 years and FFM and FFMI at the same ages.

### Variables with potential influence on telomere length

#### Boys and girls

Median LTL was not different between boys and girls at age 3 months (p = 0.48), but boys tended to have shorter LTL than girls at age 2 years (3.65 vs 3.86, p = 0.056) (Table 2). The shortening in LTL from 3 months to 2 years was not different between boys and girls (p = 0.38).

#### Parental and infant variables

Neither gestational age, parity, mode of delivery (vaginally or caesarian section), maternal pre-pregnancy BMI and weight gain during pregnancy, nor birthweight and ethnicity were associated with LTL at age 3 months, 2 years and shortening in LTL from 3 months to 2 years. Maternal and paternal age at infant’s birth did also not associate with LTL at age 3 months, 2 years and shortening in LTL from 3 months to 2 years.

#### Infant feeding

Of 145 infants, 77 were exclusively breastfed and 68 were not exclusively breastfed. Exclusively breastfed infants had longer LTL at 3 months compared to infants without exclusive breastfeeding (T/S Ratio 4.4 vs 4.1, p = 0.046), but LTL at 2 years and the shortening in LTL from 3 months to 2 years were similar in infants with exclusive BF and non-exclusive BF (p≥0.19). Duration of BF and timing of introduction of solid foods were also not correlated with LTL at 3 months, 2 years and shortening in LTL from 3 months to 2 years.

#### Length and growth

Length SDS at 3 months, 2 years and change in length SDS from 3 months to 2 years did neither associate with LTL at these ages, nor with the shortening in LTL from 3 months to 2 years.

## Discussion

Our findings in healthy, term-born infants show that 8.5% of shortening of LTL occurs from age 3 months to 2 years. The shortening in LTL from 3 months to 2 years was associated with FM%, FMI and visceral FM at age 2 years. LTL shortening during the first 2 years of life tended to associate with the gain in FM% from 3 to 6 months, within the critical window for adiposity programming.

The period from conception until age 2 years, the first 1000 days of life, is an important period for infant development [13]. Prenatal exposure to damaging environmental factors and maternal stress have been associated with shorter LTL in newborns [25, 26]. Leukocyte telomere length is one the biomarkers of aging [27, 28], next to other biomarkers like oxidative stress, inflammation and aberrations in protein and lipid metabolism, which could also affect aging rate [28]. To our knowledge, however, a very limited number of studies describe longitudinal data on LTL during infancy as most studies have used neonatal cord blood to investigate LTL at birth [15, 16] or investigated LTL cross-sectionally [17, 29]. We investigated longitudinal values for LTL at age 3 months and 2 years based on a large group of healthy, term-born infants and show that LTL decreases considerably during the first 2 year of life. There is one other paper about the change in LTL from infancy to age 2 and 3 years [12]. Our findings are in line in describing an impressive decline in LTL during the first 2 years of life. However, in contrast to the study by Bosquet Enlow et al., our first LTL measurement took place at age 3 months, thus during the critical window for adiposity programming from birth to age 6 months [7, 8], while they measured LTL for the first time at a mean age of 8.6 months.

Our findings show that LTL decreases with 8.5% from age 3 months to 2 years, which is a decline of 4.9% per year. In our PROGRAM study, LTL was investigated at age 21 years in 284 healthy, term-born subjects in the same laboratory using the same technique [18]. These subjects met the same inclusion criteria as the healthy, term-born infants of present study. We showed that LTL declined with 13.3% from age 2 years to 21 years, which is 0.7% per year after infancy, indicating that telomeres might shorten more during the first 2 years of life compared to the period from age 2 years to 21 years. Longitudinal studies from birth to 21 years have to confirm the abovementioned decline in the same subjects instead of comparing two cohorts of healthy, term-born subjects, but our findings are in line with the study by Bosquet Enlow et al. describing a stable LTL from age 2 until 3 years [12].

The shortening in LTL from age 3 months to 2 years associated significantly with FM% and FMI at age 2 years, indicating that infants with more adiposity at age 2 years had more shortening of telomeres in the period from 3 months to 2 years. Shortening in LTL from age 3 months to 2 years also associated with abdominal visceral FM at age 2 years. This is in line with a study in healthy children and adults showing that those with higher total and abdominal adiposity have lower telomere length [30]. More visceral FM has been associated with unfavorable metabolic health profiles [31, 32] and we now show that shortening in LTL during infancy is associated with visceral FM at age 2 years.

As we have shown that particularly more gain in FM% SDS from 3 to 6 months, the critical window for adiposity programming, was associated with a higher FM% at age 2 years [10], we investigated whether the shortening in LTL in the first 2 years was associated with the gain in FM% from 3 to 6 months. We found that the shortening in LTL from 3 months to 2 years tended to associate with the gain in FM% from 3 to 6 months, which could indicate that a higher gain in FM% in the critical window for adiposity programming might accelerate the shortening of LTL. It has been reported that early life adiposity programming could potentially be an early life stressor by inducing oxidative stress, which would accelerate telomere shortening [33, 34]. Also early onset of obesity has been associated with shorter LTL in children at a mean age of 11 years [35]. Our findings suggest that adverse adiposity programming in early life could contribute to more shortening of LTL.

Shortening in LTL from 3 months to 2 years also associated with FFMI at 3 months, but not at 2 years. Longer telomeres at birth have been associated with more lean mass during late infancy [15], which is in line with our results. We have, however, no data on LTL at birth and were therefore not able to study LTL and FFMI from birth onwards.

LTL was similar in boys and girls at 3 months, which has been reported in newborns [36, 37]. At 2 years, however, boys tended to have shorter LTL compared to girls, which is in line with findings during infancy, childhood and adulthood, when females have longer telomeres [12, 17, 38]. For final conclusions about sex differences in LTL, more research is required in a larger study group.

No correlations were found between LTL and birthweight and gestational age in our large group of term-born, mainly appropriate-for-gestational age infants, which is in contrast to a study describing lower birthweight result in lower cord blood LTL [39]. This study, however, also included premature infants where we only included term-born infants and in addition most of the infants in our study had a birthweight between -2 and +2 SDS. There was no correlation between cord blood LTL and gestational age, similar to our findings. Our findings are also in line with studies investigating LTL in healthy subjects at 11 years [40] and at adult age [11]. Shorter LTL has mainly been found in children born with very low birthweight [41] or born prematurely [18].

Maternal pre-pregnancy BMI did not correlate with infant LTL. This is in contrast to a study describing an association between maternal pre-pregnancy BMI and shorter newborn LTL. We, however, investigated LTL at age 3 months and 2 years instead of birth. Future research is needed to investigate pre-pregnancy BMI and LTL in different pre-pregnancy BMI classes. Maternal and paternal age did not associate with LTL until age 2 years, which is in line with literature [12].

The strength of this study is the collection of longitudinal blood samples for investigating LTL in combination with the longitudinal body composition measurements in healthy infants until the age of 2 years. We did not adjust for social economic status (SES) as we and others have shown that SES did not associate with LTL at any age [12, 18, 42]. The effect of SES on the decline of LTL during the first 2 years of life seems therefore limited, but a definite answer would require more research.

In conclusion, we present longitudinal values of LTL and show that telomere length decreases by 8.5% from age 3 months to 2 years. Shortening in LTL during the first 2 years of life was associated with FM%, FMI and visceral FM at 2 years. LTL shortening during the first 2 years of life tended to associate with a higher gain in FM% from 3 to 6 months, suggesting that adverse adiposity programming during the critical window for adiposity programming could contribute to more LTL shortening in early life.

## Supporting information

S1 Dataset (XLSX)

## Abbreviations

ADP: Air-displacement plethysmography
BF: Breastfeeding
BMI: Body mass index
CVD: Cardiovascular diseases
DXA: Dual energy x-ray absorptiometry
FFM: Fat-free mass
FFM%: Fat-free mass percentage
FFMI: Fat-free mass index
FM: Fat mass
FM%: Fat mass percentage
FMI: Fat mass index
IQR: Interquartile range
LTL: Leukocyte telomere length
T/S ratio: Telomere to single-gene copy ratio

## References

[1] Blackburn EH, Epel ES, Lin J. Human telomere biology: A contributory and interactive factor in aging, disease risks, and protection. Science. 2015;350(6265):1193–8. 10.1126/science.aab3389 26785477

[2] Codd V, Nelson CP, Albrecht E, Mangino M, Deelen J, Buxton JL, et al Identification of seven loci affecting mean telomere length and their association with disease. Nat Genet. 2013;45(4):422–7, 7e1–2. 10.1038/ng.2528 23535734 PMC4006270

[3] Zhan Y, Hagg S. Telomere length and cardiovascular disease risk. Curr Opin Cardiol. 2019;34(3):270–4. 10.1097/HCO.0000000000000613 30747731

[4] Price LH, Kao HT, Burgers DE, Carpenter LL, Tyrka AR. Telomeres and early-life stress: an overview. Biol Psychiatry. 2013;73(1):15–23. 10.1016/j.biopsych.2012.06.025 22831981 PMC3495091

[5] Monteiro PO, Victora CG. Rapid growth in infancy and childhood and obesity in later life—a systematic review. Obes Rev. 2005;6(2):143–54. 10.1111/j.1467-789X.2005.00183.x 15836465

[6] Ekelund U, Ong K, Linne Y, Neovius M, Brage S, Dunger DB, et al Upward weight percentile crossing in infancy and early childhood independently predicts fat mass in young adults: the Stockholm Weight Development Study (SWEDES). Am J Clin Nutr. 2006;83(2):324–30. 10.1093/ajcn/83.2.324 16469991

[7] Ekelund U, Ong KK, Linne Y, Neovius M, Brage S, Dunger DB, et al Association of weight gain in infancy and early childhood with metabolic risk in young adults. J Clin Endocrinol Metab. 2007;92(1):98–103. 10.1210/jc.2006-1071 17032722

[8] Leunissen RW, Kerkhof GF, Stijnen T, Hokken-Koelega A. Timing and tempo of first-year rapid growth in relation to cardiovascular and metabolic risk profile in early adulthood. JAMA. 2009;301(21):2234–42. 10.1001/jama.2009.761 19491185

[9] Woo Baidal JA, Locks LM, Cheng ER, Blake-Lamb TL, Perkins ME, Taveras EM. Risk Factors for Childhood Obesity in the First 1,000 Days: A Systematic Review. Am J Prev Med. 2016;50(6):761–79. 10.1016/j.amepre.2015.11.012 26916261

[10] de Fluiter KS, van Beijsterveldt IALP, Breij LM, Acton D, Hokken-Koelega ACS. Association Between Fat Mass in Early Life and Later Fat Mass Trajectories—Accepted—Published Online August 2020. JAMA Pediatr. 2020. 10.1001/jamapediatrics.2020.2673 PMC7432277 32804197

[11] Kajantie E, Pietilainen KH, Wehkalampi K, Kananen L, Raikkonen K, Rissanen A, et al No association between body size at birth and leucocyte telomere length in adult life—evidence from three cohort studies. Int J Epidemiol. 2012;41(5):1400–8. 10.1093/ije/dys127 22984146

[12] Bosquet Enlow M, Kane-Grade F, De Vivo I, Petty CR, Nelson CA. Patterns of change in telomere length over the first three years of life in healthy children. Psychoneuroendocrinology. 2020;115:104602 10.1016/j.psyneuen.2020.104602 32120019 PMC7183438

[13] Pietrobelli A, Agosti M, MeNu G. Nutrition in the First 1000 Days: Ten Practices to Minimize Obesity Emerging from Published Science. Int J Environ Res Public Health. 2017;14(12):1491 10.3390/ijerph14121491 29194402 PMC5750909

[14] Bijnens EM, Zeegers MP, Derom C, Martens DS, Gielen M, Hageman GJ, et al Telomere tracking from birth to adulthood and residential traffic exposure. BMC Med. 2017;15(1):205 10.1186/s12916-017-0964-8 29157235 PMC5697215

[15] de Zegher F, Diaz M, Lopez-Bermejo A, Ibanez L. Recognition of a sequence: more growth before birth, longer telomeres at birth, more lean mass after birth. Pediatr Obes. 2017;12(4):274–9. 10.1111/ijpo.12137 27071945

[16] Martens DS, Cox B, Janssen BG, Clemente DBP, Gasparrini A, Vanpoucke C, et al Prenatal Air Pollution and Newborns’ Predisposition to Accelerated Biological Aging. JAMA Pediatr. 2017;171(12):1160–7. 10.1001/jamapediatrics.2017.3024 29049509 PMC6233867

[17] Skilton MR, Nakhla S, Ayer JG, Harmer JA, Toelle BG, Leeder SR, et al Telomere length in early childhood: Early life risk factors and association with carotid intima-media thickness in later childhood. Eur J Prev Cardiol. 2016;23(10):1086–92. 10.1177/2047487315607075 26405259

[18] Smeets CC, Codd V, Samani NJ, Hokken-Koelega AC. Leukocyte Telomere Length in Young Adults Born Preterm: Support for Accelerated Biological Ageing. PLoS One. 2015;10(11):e0143951 10.1371/journal.pone.0143951 26619005 PMC4664383

[19] Nakamura K, Ishikawa N, Izumiyama N, Aida J, Kuroiwa M, Hiraishi N, et al Telomere lengths at birth in trisomies 18 and 21 measured by Q-FISH. Gene. 2014;533(1):199–207. 10.1016/j.gene.2013.09.086 24080483

[20] Breij Laura M, Kerkhof Gerthe F, De Lucia Rolfe E, Ong Ken K, Abrahamse-Berkeveld M, Acton D, et al Longitudinal fat mass and visceral fat during the first 6 months after birth in healthy infants: support for a critical window for adiposity in early life. Pediatric Obesity. 2017;12(4):286–94. 10.1111/ijpo.12139 27072083 PMC6186414

[21] COSMED. Pea Pod Brochure ENGLISH

[22] de Fluiter KS, van Beijsterveldt IALP, Goedegebuure WJ, Breij LM, Spaans AMJ, Acton D, et al Longitudinal body composition assessment in healthy term-born infants until 2 years of age using ADP and DXA with vacuum cushion. Eur J Clin Nutr. 2020;74(4):642–50. 10.1038/s41430-020-0578-7 32055012

[23] De Lucia Rolfe E, Modi N, Uthaya S, Hughes IA, Dunger DB, Acerini C, et al Ultrasound Estimates of Visceral and Subcutaneous-Abdominal Adipose Tissues in Infancy. Journal of Obesity. 2013;2013:951954 10.1155/2013/951954 23710350 PMC3654330

[24] Cawthon RM. Telomere measurement by quantitative PCR. Nucleic Acids Res. 2002;30(10):e47 10.1093/nar/30.10.e47 12000852 PMC115301

[25] Send TS, Gilles M, Codd V, Wolf I, Bardtke S, Streit F, et al Telomere Length in Newborns is Related to Maternal Stress During Pregnancy. Neuropsychopharmacology. 2017;42(12):2407–13. 10.1038/npp.2017.73 28397798 PMC5645750

[26] Wai KM, Umezaki M, Kosaka S, Mar O, Umemura M, Fillman T, et al Impact of prenatal heavy metal exposure on newborn leucocyte telomere length: A birth-cohort study. Environ Pollut. 2018;243(Pt B):1414–21. 10.1016/j.envpol.2018.09.090 30278415

[27] Xia X, Chen W, McDermott J, Han J-DJ. Molecular and phenotypic biomarkers of aging. F1000Res. 2017;6:860-. 10.12688/f1000research.10692.1 28663789 PMC5473407

[28] Engelfriet PM, Jansen EHJM, Picavet HSJ, Dollé MET. Biochemical markers of aging for longitudinal studies in humans. Epidemiol Rev. 2013;35(1):132–51. 10.1093/epirev/mxs011 23382477 PMC4707878

[29] Rufer N, Brümmendorf TH, Kolvraa S, Bischoff C, Christensen K, Wadsworth L, et al Telomere Fluorescence Measurements in Granulocytes and T Lymphocyte Subsets Point to a High Turnover of Hematopoietic Stem Cells and Memory T Cells in Early Childhood. J Exp Med. 1999;190(2):157–68. 10.1084/jem.190.2.157 10432279 PMC2195579

[30] Lee M, Martin H, Firpo MA, Demerath EW. Inverse association between adiposity and telomere length: The Fels Longitudinal Study. Am J Hum Biol. 2011;23(1):100–6. 10.1002/ajhb.21109 21080476 PMC3245638

[31] Ferreira AP, da Silva Junior JR, Figueiroa JN, Alves JG. Abdominal subcutaneous and visceral fat thickness in newborns: correlation with anthropometric and metabolic profile. J Perinatol. 2014;34(12):932–5. 10.1038/jp.2014.110 24901453

[32] Gishti O, Gaillard R, Durmus B, Abrahamse M, van der Beek EM, Hofman A, et al BMI, total and abdominal fat distribution, and cardiovascular risk factors in school-age children. Pediatr Res. 2015;77(5):710–8. 10.1038/pr.2015.29 25665058

[33] von Zglinicki T. Oxidative stress shortens telomeres. Trends Biochem Sci. 2002;27(7):339–44. 10.1016/s0968-0004(02)02110-2 12114022

[34] Valdes AM, Andrew T, Gardner JP, Kimura M, Oelsner E, Cherkas LF, et al Obesity, cigarette smoking, and telomere length in women. Lancet. 2005;366(9486):662–4. 10.1016/S0140-6736(05)66630-5 16112303

[35] Buxton JL, Walters RG, Visvikis-Siest S, Meyre D, Froguel P, Blakemore AIF. Childhood obesity is associated with shorter leukocyte telomere length. The Journal of clinical endocrinology and metabolism. 2011;96(5):1500–5. 10.1210/jc.2010-2924 21349907 PMC3137462

[36] Okuda K, Bardeguez A, Gardner JP, Rodriguez P, Ganesh V, Kimura M, et al Telomere length in the newborn. Pediatr Res. 2002;52(3):377–81. 10.1203/00006450-200209000-00012 12193671

[37] Entringer S, Epel ES, Lin J, Buss C, Shahbaba B, Blackburn EH, et al Maternal psychosocial stress during pregnancy is associated with newborn leukocyte telomere length. Am J Obstet Gynecol. 2013;208(2):134 e1–7. 10.1016/j.ajog.2012.11.033 23200710 PMC3612534

[38] Barrett ELB, Richardson DS. Sex differences in telomeres and lifespan. Aging Cell. 2011;10(6):913–21. 10.1111/j.1474-9726.2011.00741.x 21902801

[39] Lee SP, Hande P, Yeo GS, Tan EC. Correlation of cord blood telomere length with birth weight. BMC Res Notes. 2017;10(1):469 10.1186/s13104-017-2791-6 28886728 PMC5591543

[40] Slykerman RF, Joglekar MV, Hardikar AA, Satoor SN, Thompson JMD, Jenkins A, et al Maternal stress during pregnancy and small for gestational age birthweight are not associated with telomere length at 11years of age. Gene. 2019;694:97–101. 10.1016/j.gene.2019.01.017 30738962

[41] Raqib R, Alam DS, Sarker P, Ahmad SM, Ara G, Yunus M, et al Low birth weight is associated with altered immune function in rural Bangladeshi children: a birth cohort study. Am J Clin Nutr. 2007;85(3):845–52. 10.1093/ajcn/85.3.845 17344508

[42] Smeets CCJ, Codd V, Denniff M, Samani NJ, Hokken-Koelega ACS. Effects of size at birth, childhood growth patterns and growth hormone treatment on leukocyte telomere length. PloS one. 2017;12(2):e0171825–e. 10.1371/journal.pone.0171825 28178350 PMC5298325

